# Dynamics of cardiovascular and baroreflex readjustments during a light-to-moderate exercise transient in humans

**DOI:** 10.1007/s00421-022-05011-4

**Published:** 2022-07-21

**Authors:** Anna Taboni, Nazzareno Fagoni, Timothée Fontolliet, Giovanni Vinetti, Guido Ferretti

**Affiliations:** 1 Department of Anaesthesiology, Pharmacology, Intensive Care, and Emergencies, University of Geneva, Geneva, Switzerland; 2 Department of Molecular and Translational Medicine, University of Brescia, Viale Europa 11, 25123 Brescia, Italy; 3 AAT Brescia, Department of Anaesthesiology, Intensive Care and Emergency Medicine, Spedali Civili University Hospital, Brescia, Italy; 4 Institute of Mountain Emergency Medicine, Eurac Research, Bolzano, Italy

**Keywords:** Vagal withdrawal, Sequence method, Arterial baroreflex, Baroreflex resetting, Cardiac output, Heart rate

## Abstract

**Purpose:**

We hypothesised that, during a light-to-moderate exercise transient, compared to an equivalent rest-to-exercise transient, (1) a further baroreflex sensitivity (BRS) decrease would be slower, (2) no rapid heart rate (HR) response would occur, and (3) the rapid cardiac output (CO) response would have a smaller amplitude (*A*1). Hence, we analysed the dynamics of arterial baroreflexes and the HR and CO kinetics during rest-to-50 W (0–50 W) and 50-to-100 W (50–100 W) exercise transients.

**Methods:**

10 subjects performed three 0–50 W and three 50–100 W on a cycle ergometer. We recorded arterial blood pressure profiles (photo-plethysmography) and R-to-R interval (RRi, electrocardiography). The former were analysed to obtain beat-by-beat mean arterial pressure (MAP) and stroke volume (SV). CO was calculated as SV times HR. BRS was measured by modified sequence method.

**Results:**

During 0–50 W, MAP transiently fell (− 9.0 ± 5.7 mmHg, *p* < 0.01) and BRS passed from 15.0 ± 3.7 at rest to 7.3 ± 2.4 ms mmHg^−1^ at 50 W (*p* < 0.01) promptly (first BRS sequence: 8.1 ± 4.6 ms mmHg^−1^, *p* < 0.01 vs. rest). During 50–100 W, MAP did not fall and BRS passed from 7.2 ± 2.6 at 50 W to 3.3 ± 1.3 ms mmHg^−1^ at 100 W (*p* < 0.01) slowly (first BRS sequence: 5.3 ± 3.1 ms mmHg^−1^, *p* = 0.07 vs. 50 W). A1 for HR was 9.2 ± 6.0 and 6.0 ± 4.5 min^−1^ in 0–50 W and 50–100 W, respectively (*p* = 0.19). The corresponding A1 for CO were 2.80 ± 1.54 and 0.91 ± 0.55 l∙min^−1^ (*p* < 0.01).

**Conclusion:**

During 50–100 W, with respect to 0–50 W, BRS decreased more slowly, in absence of a prompt pressure decrease. BRS decrease and rapid HR response in 50–100 W were unexpected and ascribed to possible persistence of some vagal tone at 50 W.

**Supplementary Information:**

The online version contains supplementary material available at 10.1007/s00421-022-05011-4.

## Introduction

Exercise requires a tight control of arterial blood pressure to provide an optimal cerebral perfusion. This control is provided by several mechanisms, the most important being the arterial baroreflex. Although the arterial baroreflex has been widely investigated during exercise (Potts et al. 1993; Papelier et al. 1994; Iellamo et al. 1997; Norton et al. 1999; Ogoh et al. 2005; Peçanha et al. 2017; Porta et al. 2018), little is known about its responses during the exercise transients. When the neck suction-neck pressure technique is applied at the exercise steady-state, a shift of the entire baroreflex logistic curve upward and rightward (baroreflex resetting) and a displacement of the operating point (OP) toward the baroreflex threshold are observed (Ogoh et al. 2005; Raven et al. 2006). When the sequence method is applied instead, a decrease of spontaneous baroreflex sensitivity (BRS) around OP appears, coherently with the observed displacement of the OP toward the baroreflex threshold with the aforementioned technique. Moreover, the baroreflex resetting appears as a displacement of the baroreflex sequences, and thus OP, toward higher values of both pressure and heart rate (HR) with respect to rest (Rowell and O’Leary 1990; Iellamo et al. 1997; Raven et al. 2006; Porta et al. 2018).

Different mechanisms have been called upon as determinants of the baroreflex alterations at exercise: a feedforward central command, and feedback loops such as the exercise pressor reflex (Ogoh et al. 2002, 2007; Fadel et al. 2003; Gallagher et al. 2006; Raven et al. 2006). More recently, Bringard et al. (Potts et al. 1993; Iellamo et al. 1997; Norton et al. 1999; Raven et al. 2006; Vallais et al. 2009; Bringard et al. 2017; Fontolliet et al. 2018) analysed the dynamics of baroreflex resetting upon exercise onset with a closed-loop approach. They found that, as exercise started, the BRS decreased almost immediately, whereas OP resetting was slower and delayed. Bringard et al. (Potts et al. 1993; Iellamo et al. 1997; Norton et al. 1999; Raven et al. 2006; Vallais et al. 2009; Bringard et al. 2017; Fontolliet et al. 2018) interpreted the former result as being an effect of sudden withdrawal of vagal tone (Fagraeus and Linnarsson 1976; Lador et al. 2006) when exercise begins, and concluded that their results were compatible with a central command mechanism. Their interpretation is coherent with the subsequent demonstration of very low BRS under parasympathetic blockade with atropine (Fontolliet et al. 2018).

The hypothesis of vagal withdrawal stemmed from experiments in which the kinetics of the rapid component (phase I) of the HR response at exercise onset was analysed (Fagraeus and Linnarsson 1976). In fact, at the very beginning of exercise, HR increases abruptly at first, the so-called phase I, and then more slowly. When the vagal activity was blocked with atropine, the phase I was completely abolished and only a slow HR increase was observed (Fagraeus and Linnarsson 1976). The vagal withdrawal hypothesis could at least partially explain also the phase I of cardiac output (CO) responses to exercise onset, i.e., the rapid initial response (Lador et al. 2006). Further results, obtained during experiments in acute hypoxia (Lador et al. 2008), under lower body negative pressure condition (Fagoni et al. 2020), and particularly during vagal blockade with atropine (Fontolliet et al. 2021), were also compatible with the hypothesis.

If the vagal withdrawal hypothesis was correct, then we would expect that during a light-to-moderate exercise transient, with respect to an equivalent rest-to-exercise transient, (1) a further BRS decrease at increasing workload would not be immediate, (2) no phase I HR response would occur, and (3) the phase I CO response would have a smaller amplitude, the size of which would be determined by the size of SV response only. The aim of the present study is to test the above hypothesis, by analysing the dynamics of arterial baroreflexes (Bringard et al. 2017) and the HR and CO kinetics (Lador et al. 2006; Fontolliet et al. 2021) during the rest-to-50 W and the 50-to-100 W exercise transients.

## Materials and methods

### Subjects

Ten healthy, physically active subjects were enlisted (9 males and 1 female). Age, height, and body mass were 31 ± 6 years, 174 ± 9 cm, and 69 ± 11 kg, respectively. Their maximal oxygen consumption measured during a ramp test was 3.3 ± 0.4 l min^−1^. None reported history of cardiovascular, pulmonary, or neurological diseases; none was taking medications or was pregnant at the time of the study. The subjects were asked to refrain from drinking coffee or from smoking for 24 h before the experiments. All subjects gave their informed consent after having received a detailed description of the methods and experimental procedures of the study. Every subject was aware of the right of withdrawing from the study at any time without jeopardy. This study was performed in line with the principles of the Declaration of Helsinki. Approval was granted by the Commission Cantonale d’Éthique de la Recherche, Canton de Genève, CH (Date 11th July 2018-No. 2018-00913).

### Experimental procedure

**Fig. 1:**
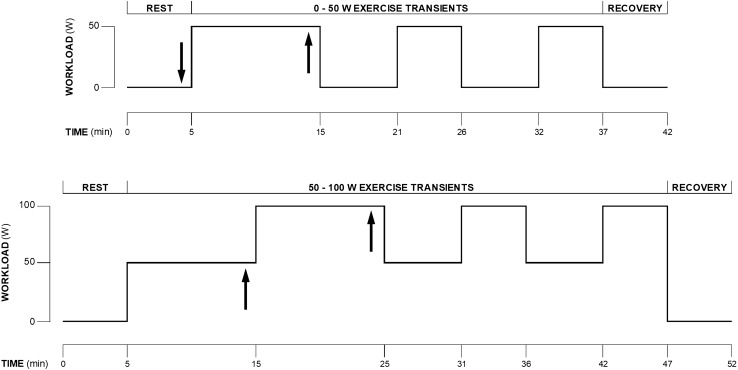
The experimental protocol comprised two sessions: one for the analysis of the 0 to 50 W transient and one for the 50 to 100 W transient. Arrows indicate the time at which a 20 µl capillary blood sample was taken from an earlobe for measuring blood lactate concentration

The subjects came to the laboratory on one occasion, at least 2 h after a light meal. Room temperature was 24 ± 2 °C. After instrumentation and wearing cycling shoes, the subject took place on an electromagnetically braked cycle ergometer (Lode Corival, Lode B.V., Groningen, The Netherlands). The experimental protocol comprised two sessions, one for rest to 50 W transients (0–50 W) and one for 50-to-100 W transients (50–100 W), which were administered in random order and were separated by 2–3 h, to allow resting, drinking, and a light meal or snacks. The two experimental sessions are depicted in Fig. 1. Both 0–50 W and 50–100 W sessions consisted of three trials to obtain three repetitions of the two exercise transients to be superimposed to decrease the signal-to-noise ratio.

In the 0–50 W session, after calibration of the instruments with the subject quietly resting on the cycle ergometer, 5 min of rest were recorded. At the 4th min, a 20 µl capillary blood sample was taken from an earlobe for blood lactate concentration [(La)] measurement. Afterwards, the subject started to pedal at 50 W at 60 rpm (trial 1). The subject continued to exercise for 10 min to reach and ensure at least 5 min of steady-state condition (Ferretti et al. 2017); a 20 µl capillary blood sample was taken from an earlobe for [La] measurement at the 9th min. At the end of this trial, the subject underwent further 6 min of resting recovery, followed by 5 min exercising at 50 W (trial 2). This same sequence was repeated for trial 3. At the end of trial 3, 5 min resting was allowed. During 0–50 W, the flywheel was not pre-accelerated; this usually implies that the mechanical power necessary for the flywheel acceleration may compensate for the delayed activation of the magnetic brake of the cycle ergometer (Hibi et al. 1996).

In the 50–100 W session, after calibration of the instruments with the subject quietly resting on the cycle ergometer, 5 min of rest was recorded. Then, the subject pedalled at 50 W at 60 rpm for 10 min, to ensure at least 5 min of steady state condition (Ferretti et al. 2017); a 20 µl capillary blood sample was taken from an earlobe for [La] measurement at the 9th min. Afterwards, the workload was increased to 100 W for 10 min for the same reasons explained above and at the 9th min [La] was measured (trial 1). Afterwards, the subject continued pedalling for further 6 min at 50 W, followed by 5 min at 100 W (trial 2). This same sequence was repeated for trial 3. At the end of trial 3, 5 min resting was allowed.

### Measurements

Continuous non-invasive arterial blood pressure profiles were recorded at the medium finger of the left arm (Portapres, Finapres^®^ Medical Systems, Enschede, The Netherlands). The left arm was positioned on a rigid support at the heart level. Beat-by-beat HR was recorded by electrocardiography (ECG100C module, BIOPAC^®^ Systems Inc., Goleta, CA, USA). Applied workload and pedalling speed were continuously monitored by analogic outputs from the cycle ergometer which were digitalised (UIM100C module, BIOPAC^®^ Systems Inc., Goleta, CA, USA) and used to provide a precise time alignment of the three repetitions of the two exercise transients. All signals were collected and sampled at 400 Hz (MP150 system with AcqKnowledge acquisition and analysis software, BIOPAC^®^ Systems Inc., Goleta, CA, USA) and stored on a personal computer for subsequent analysis. [La] was measured by an enzymatic-amperometric method (Biosen C-Line Glucose and Lactate analyser, EKF Diagnostics, Cardiff, UK) on 20 μl capillary blood samples.

### Data treatment

Arterial blood pressure profiles were analysed to obtain beat-by-beat values of systolic (SAP), diastolic (DAP), and mean (MAP) blood arterial pressure using the Beatscope^®^ software (Finapres^®^ Medical Systems, Enschede, The Netherlands). The same software provided a beat-by-beat calculation of the stroke volume (SV) through the Modelflow method (Wesseling et al. 1993). Beat-by-beat CO was calculated as the SV times the corresponding HR and total peripheral resistances (TPR) as the ratio between MAP and CO.

The BRS during steady states was calculated with the sequence method (Bertinieri et al. 1988) using MAP and R-to-R interval (RRi) as independent and dependent variable, respectively (Taboni et al. 2018). A phase shift of one beat between MAP and RRi was introduced (Steptoe and Vogele 1990), and then, sequences of three or more consecutive beats characterised by consensual increase or decrease in MAP and RRi were identified. Within each sequence, the relationship between RRi and MAP was analysed by linear regression to compute the slope and the coefficient of determination (*R*^2^). When *R*^2^ > 0.85, the slope was retained (Iellamo et al. 1997). In steady-state conditions, the mean slope of the RRi versus MAP relationship was considered representative of the BRS for each subject and the mean RRi and MAP value was considered as the corresponding OP.

For 0–50 W, data in steady-state conditions were computed on the last 5 min of the initial resting period and of the 10-min-lasting 50 W exercise bout. For 50–100 W, data in steady-state conditions were computed on the last 5 min of the 10-min-lasting 50 W and 100 W exercise bouts.

The baroreflex patterns during 0–50 W and 50–100 W were analysed with the same approach as previously proposed (Bringard et al. 2017). Briefly, at the very beginning of a rest-to-exercise transient, MAP decreases and successively recovers, and the minimum value during this initial MAP readjustment was retained and defined as minimum MAP. Then, the BRS was calculates as follows: when at least three consecutive beats were characterised by consensual increase or decrease of MAP and RRi, these beats were treated as baroreflex sequences on which the sequence method could be applied, as described above.

Dynamics of the CO and HR changes over time (*f*_(t)_) during the two exercise transients were analysed through bi exponential model (Barstow and Molé 1987; Lador et al. 2006)

1 $$\begin{gathered} f_{\left( t \right)} = b + A_{1} \left( {1 - e^{{ - \frac{t}{{\tau_{1} }}}} } \right) + H_{{\left( {t - d} \right)}} A_{2} \left( {1 - e^{{\frac{d - t}{{\tau_{2} }}}} } \right) \hfill \\ H_{{\left( {t - d} \right)}} = \left\{ {\begin{array}{*{20}c} {0,\, t - d < 0} \\ {1,\, t - d \ge 0} \\ \end{array} } \right., \hfill \\ \end{gathered}$$

where *b* is the baseline, A is the amplitude, *d* is the time delay between the two components, and τ is the time constant. The subscripts 1 and 2 refer to the initial (phase I) and the primary components, respectively. For every subject, the three repetitions were time aligned at exercise onset or workload increase. When the amplitude of one phase resulted equal to 0 l min^−1^ for CO or 0 bpm for HR, the corresponding time constant was not considered for the statistical analysis.

### Statistical analysis

Data are presented as mean ± standard deviation. Student *T* test for paired samples was used to compare pre- with post-transient steady-state data in the two experimental conditions. One-way ANOVA for repeated measures was used to investigate differences among MAP and BRS measured at different time points during exercise transients. Tukey’s multiple comparisons test was used to isolate differences when necessary. Differences were considered significant when *p* < 0.05; otherwise, they were considered non-significant. The statistical software Prism (version 8, GraphPad^®^, La Jolla, CA, USA) was used. Data fitting with Eq. 1 was performed on the superimposition of the three repetitions for each exercise transient for each subject; MATLAB (version 9.5.0.944444 with Curve Fitting Toolbox, The MathWorks, Inc., Natick, MA, USA) was used for this aim. The resulting Eq. 1 parameters obtained in the two exercise transients were compared by Student *T* test.

## Results

The steady-state cardiovascular parameters, BRS, and [La] are reported in Table 1. The 50 W steady-state data did not differ between the two protocols. When the 50 W steady state was compared to the corresponding resting data, all cardiovascular parameters resulted different, except for DAP. When the 100 W was compared to the corresponding 50 W steady state, all cardiovascular parameters resulted different, except for DAP and SV. Moreover, the BRS at steady state decreased with increasing the workload.

**Table 1:** Steady-state values in the four experimental conditions

	0–50 W transient			50–100 W transient		
	Rest	50 W	*p* Value	50 W	100 W	*p* Value
SAP (mmHg)	121 ± 10	147 ± 9	< 0.0001	154 ± 15	170 ± 29	0.0152
DAP (mmHg)	68 ± 13	69 ± 10	0.4494	74 ± 15	76 ± 17	0.2061
MAP (mmHg)	83 ± 12	91 ± 9	0.0024	95 ± 15	100 ± 19	0.0348
HR (min^−1^)	78 ± 9	96 ± 9	< 0.0001	96 ± 10	122 ± 20	< 0.0001
RRi (ms)	789 ± 90	636 ± 67	0.0001	634 ± 70	506 ± 79	< 0.0001
SV (ml)	75 ± 20	102 ± 24	< 0.0001	94 ± 25	97 ± 21	0.5684
CO (l min^−1^)	5.9 ± 1.9	9.7 ± 2.4	< 0.0001	9.0 ± 2.2	11.6 ± 2.0	0.0006
TPR (mmHg min l^−1^)	16.0 ± 5.8	10.0 ± 2.8	0.0002	11.5 ± 3.7	9.3 ± 2.7	0.0004
BRS (ms mmHg^−1^)	15.0 ± 3.7	7.3 ± 2.4	0.0006	7.2 ± 2.6	3.3 ± 1.3	0.0001
[La] (mmol l^−1^)	1.24 ± 0.33	1.24 ± 0.72	0.9956	1.79 ± 1.00	2.13 ± 1.24	0.2489

*SAP* systolic arterial pressure, *DAP* diastolic arterial pressure, *MAP* mean arterial pressure, *HR* heart rate, *RRi* R-to-R interval, *SV* stroke volume, *CO* cardiac output, *TPR* total peripheral resistances, *BRS* baroreflex sensitivity, [La] capillary blood lactate concentration

**Fig. 2:**
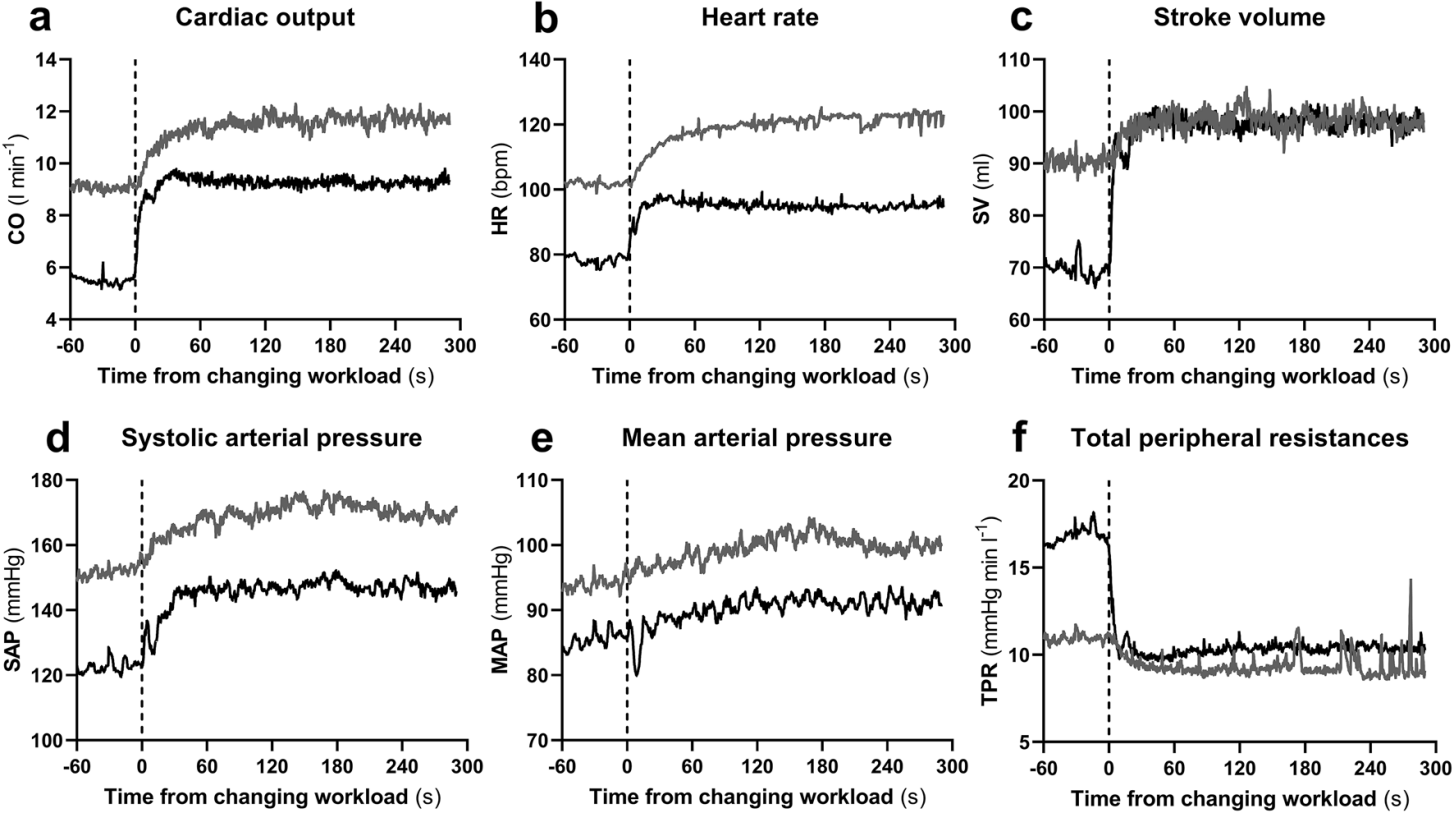
Time course of the cardiac output (CO), heart rate (HR), stroke volume (SV), systolic and mean arterial pressure (SAP and MAP, respectively), and total peripheral resistances (TPR) during the rest to 50 W (black line) and the 50 to 100 W (grey line) exercise transients. Mean values from all subjects. The time scale represents the time elapsed since the change of the workload

The time course of the main cardiovascular parameters is shown in Fig. 2. During 50–100 W (grey line), with respect to 0–50 W (black line), CO and HR increased less sharply (a, b, respectively), SV showed a smaller increase (c), and TPR decreased slowly and by a lesser amount (f). Moreover, during 0–50 W, SAP showed an initial notch (d) which corresponded to an MAP transient fall (e). This fall of MAP was not observed in 50–100 W.

Parameters obtained from the analysis of the CO kinetics are reported in Table 2. Compared to 0-to-50 W, 50–100 W was characterised by higher *b*, smaller *A*_1_, higher τ_1_, similar *A*_2_, and higher τ_2_. Furthermore, in 0–50 W and 50–100 W, *A*_1_ accounted for 69 ± 23% and 35 ± 19% of the total amplitude, respectively (*p* = 0.0022). Parameters obtained from the analysis of the HR kinetics are reported in Table 3. In this case, both *A*_1_ and *A*_2_ were similar in the two investigated transients, whereas *b*, τ_1_, and τ_2_ resulted higher in 50–100 W than in 0–50 W. Additionally, *A*_1_ accounted for the 55 ± 26% and the 33 ± 24% of the total amplitude in 0–50 W and 50–100 W, respectively (*p* = 0.0593). Of note, the wide standard deviation observed for τ_1_ and τ_2_ results from a subject, whose data are more than 2 SD far from the mean value, and thus who can be considered an outlier (see Table S2 on Supplementary material). After exclusion of this subject, τ_1_ and τ_2_ for HR turn out equal to 0.9 ± 1.0 s and 2.9 ± 2.0 s, respectively.

**Table 2:** Mean ± standard deviation of the parameters of the cardiac output kinetics as obtained by data fitting with Eq. 1.

	Baseline (l min^−1^)	Initial phase		Primary phase	
		Amplitude (l min^−1^)	Time constant (s)	Amplitude (l min^−1^)	Time constant (s)
Rest–50 W	5.45 ± 1.37	2.80 ± 1.54	2.1 ± 1.0	1.09 ± 0.84	10.5 ± 6.6
50–100 W	9.14 ± 1.78	0.91 ± 0.55	5.1 ± 3.2	1.74 ± 0.86	44.3 ± 30.5
*p* value	< 0.0001	0.0019	0.0108	0.1065	0.0075

Individual data are reported in the Supplementary material (Table S1)

**Table 3:** Mean ± standard deviation of the parameters of the heart rate kinetics as obtained by data fitting with Eq. 1.

	Baseline (min^−1^)	Initial phase		Primary phase	
		Amplitude (min^−1^)	Time constant (s)	Amplitude (min^−1^)	Time constant (s)
Rest–50 W	79 ± 9	9.2 ± 6.0	1.5 ± 2.1	7.6 ± 5.3	12.1 ± 27.8
50–100 W	102 ± 13	6.0 ± 4.5	5.6 ± 2.6	12.6 ± 6.3	49.1 ± 44.2
*p* value	0.0002	0.1873	0.0038	0.0714	0.0458

Individual data are reported in the Supplementary material (Table S2)

**Fig. 3:**
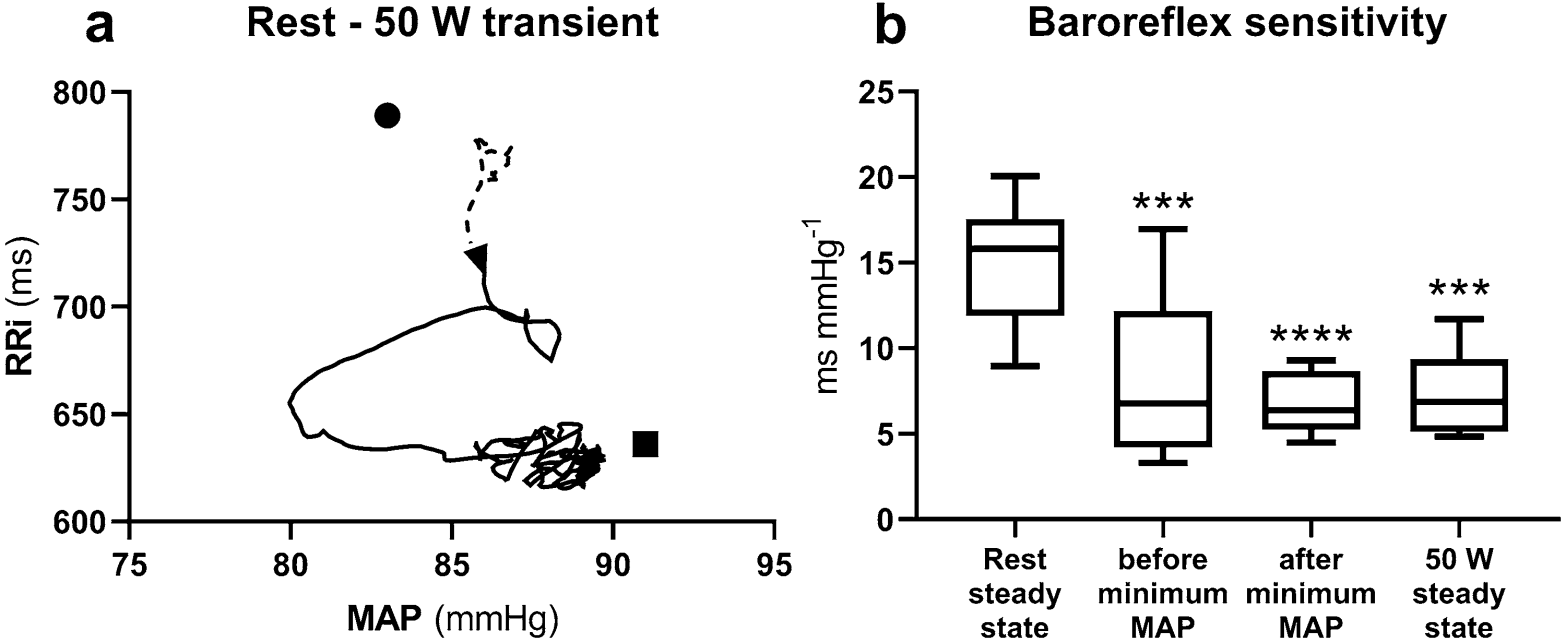
**a** Contour plot of the relationship between R-to-R interval (RRi) and mean arterial pressure (MAP) during the rest-to-50 W exercise transient. A time shift of 0.1 s was applied between MAP and RRi. Mean values from all subjects from 10 s before (dashed line) to 60 s after (continuous line) exercise onset (black arrowhead). Black dot: rest steady state; black square: 50 W steady state. **b** Tukey representation of the baroreflex sensitivity measured during the indicated conditions. *: significantly different vs. rest steady state (***p < 0.001; ****p < 0.0001)

**Fig. 4:**
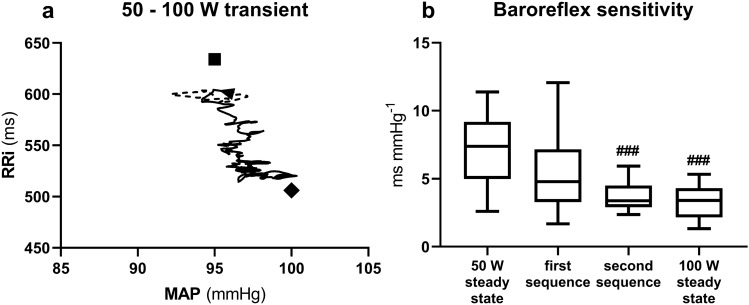
**a** contour plot of the relationship between R-to-R interval (RRi) and mean arterial pressure (MAP) during the 50 to 100 W exercise transient. A time shift of 0.1 s was applied between MAP and RRi. Mean values from all subjects from 10 s before (dashed line) to 60 s after (continuous line) workload change (black arrowhead). Black square: 50 W steady state; black diamond: 100 W steady state. **b** Tukey representation of the baroreflex sensitivity measured during the indicated conditions. #: significantly different vs. 50 W steady state (###*p* < 0.001)

During 0–50 W, MAP showed a sudden decrease at the very beginning of exercise (Fig. 2e, black line). Minimum MAP was 74 ± 11 mmHg (*p* = 0.0002 and *p* < 0.0001 vs. rest and 50 W steady state, respectively) and appeared after 8.2 ± 1.9 s from exercise onset. A similar decrease was not observed during 50–100 W (Fig. 2e, grey line). This implied that the relationship between MAP and RRi resulted different in the two transients, as shown in Figs. 3a and 4a.

In 0–50 W, during the initial MAP decrease, also RRi decreased (Fig. 3a). During this period and before, a minimum MAP was reached, it was possible to detect a BRS sequence which appeared 4.8 ± 2.2 s after exercise onset and was 3.4 ± 0.3 beats long. The measured slope of these sequences is reported in Fig. 3b, before minimum MAP. After attainment of minimum MAP, the pattern of the RRi versus MAP relationship changed, as long as a decrease in RRi was associated with an increase in MAP. The subsequent BRS sequences were identified after this second period, when approaching the steady state. The slope of the first BRS sequence observed after minimum MAP is also reported in Fig. 3b. These sequences appeared 16.1 ± 7.3 s after exercise onset.

During 50–100 W, no significant MAP decrease was detected following the workload increase (Fig. 2e, grey line and Fig. 4a). Consequently, it was not possible to identify BRS sequences which may correspond to those found during 0–50 W. Therefore, to analyse the dynamic changes in BRS, we considered the first two BRS sequences after workload increase. The first BRS sequence was characterised by consensual decrease in MAP and RRi in 22 out of 30 exercise transients and took place within 6.4 ± 5.5 s after the workload change, and the second sequence was characterised by consensual decrease in MAP and RRi in 17 out of 30 exercise transients and appeared 17.5 ± 11.5 s after the workload change. The slopes of both these BRS sequences are reported in Fig. 4b.

## Discussion

The results are in line with the tested hypothesis. As forecasted, the BRS decreased following a different pattern, and the CO kinetics had smaller *A*_1_ and slower τ_1_ during 50–100 W than during 0–50 W. Moreover, during 50–100 W, the HR showed a phase I; thus, it participates in the CO phase I, which had a similar *A*_1_ and a slower τ_1_ than during 0–50 W. This is in line with the concept that some degree of vagal activity is still present at 50 W (White and Raven 2014). We suggest that the withdrawal of the vagal tone is responsible for the HR phase I and BRS decrease both during the 0–50 W and the 50–100 W.

### Cardiovascular response kinetics

The data obtained from the analysis of the kinetics of CO and HR during 0-50 W are in line with previous findings (Lador et al. 2006, 2013; Fagoni et al. 2020; Fontolliet et al. 2021), indicating that *A*_1_ of CO results from the sudden increase of both HR and SV. The *A*_1_ of HR was proposed to have a neural origin and be mediated by rapid vagal withdrawal (Fagraeus and Linnarsson 1976; Lador et al. 2006, 2008; McNarry et al. 2014; Fontolliet et al. 2018, 2021; Fagoni et al. 2020). At variance, the prompt SV increase has been hypothesised to have mechanical origin, due to (1) a displacement of blood from the periphery toward the thorax (Chung et al. 1997; Sundblad et al. 2000, 2014; Naeije and Badagliacca 2017) and (2) a sudden decrease in TPR as a consequence of a strong vasodilation in the contracting muscles (Elstad et al. 2002), as shown in Fig. 2f. Nevertheless, neural mechanisms have also been evoked in the interpretation of the SV increase, in so far as this increase is at least partially sustained by incurring sympathetic activation, which, however, may have a response time too slow to act during the phase I (Fontolliet et al. 2021). In addition, some authors have proposed that also vagal withdrawal may contribute to the SV increase (DeGeest et al. 1965; Machhada et al. 2016).

If indeed the SV response has mechanical origin, the impact of SV on the CO response in phase I may be different in the 0–50 W from the 50–100 W. In fact, the 0–50 W starts with relaxed leg muscles; hence, the abrupt initiation of the contraction–relaxation cycles determines the activation of the muscle pump that, in turn, displaces the venous blood toward the thorax and the right heart. After passing through the pulmonary circulation, the blood arrives to the left heart thus contributing to the CO increase. Moreover, starting from rest, leg exercise is capable to increase the muscle blood flow rapidly up to fourfold (MacDonald et al. 1998). Conversely, during the 50–100 W, the muscle pump is already active and the muscle capillaries are already open, so that their effect on the CO, if any, is expected to be of a lesser amount than during the 0–50 W.

During 50–100 W, both CO and HR showed an initial phase which resulted slower than during 0–50 W (Table 3). It has been recently found (Fontolliet et al. 2021) that complete vagal blockade with atropine abolishes the phase I of the HR response to a rest-to-exercise transient, but not the phase I of the CO response. This is the best and clearest evidence supporting the hypothesis that a rapid HR response may be mediated by sudden withdrawal of the vagal tone. This being the case, the presence of an *A*_1_ of HR during 50–100 W would be in line with the concept that during exercise, at least at 50 W, some degree of vagal activity is still present (White and Raven 2014), vagal withdrawal thus being incomplete. This is coherent with previous results obtained at steady state under selective β_1_-adrenergic blockade, demonstrating that HR progressively increases with increasing workload, despite the lack of sympathetic control of the sinus node (Ferretti et al. 2005). Moreover, previous analysis of the HR variability showed that its total power, which can be considered an indirect measure of the vagal activity (Warren et al. 1997; Montano et al. 1998; Challapalli et al. 1999; Formes et al. 2005), in control conditions at exercise steady state (80 W) was higher than under vagal blockade with atropine at rest (Fontolliet et al. 2018), further sustaining the concept of an incomplete vagal withdrawal at least in the light–moderate intensity domain.

Although during 0–50 W, both HR and SV clearly contribute to the CO phase I, the results are not so straightforward for 50–100 W. In fact, if we assume that SV does not change during the initial phase of 50–100 W, the CO asymptote of the initial phase (CO_I_) should be equal to

2 $${\mathrm{CO}}_{I}={\mathrm{SV}}_{b}\left({\mathrm{HR}}_{b}+{\mathrm{HR}}_{A1}\right).$$

From the data of the present study, CO_I_ turns out equal to 9.66 ± 1.89 l min^−1^. The assumption behind Eq. 2 implies that CO_I_ should correspond to the sum of baseline CO plus *A*_1_ for CO. This last is equal to 10.05 ± 2.15 l min^−1^ (see Table S1), a value which, although similar to CO_I_, yet appears to be significantly different from it (*p* = 0.0407). In this context, the difference between these two values (~ 0.39 l min^−1^) would provide the portion of CO increase during the initial phase due to SV increase. However, if we adopt a different approach and calculate the percentage increase in CO during the initial phase, we can compare it to the percentage HR increase during the same phase. The former resulted to be 10 ± 6% and the latter 6 ± 5% (*p* = 0.1191); the difference between these two values (~ 3%), which should correspond to the percentage increase in SV during the initial phase, was not significantly different from zero. Therefore, no clear-cut conclusion can be attained concerning the lack of a role for SV as a determinant of the *A*_1_ of CO in 50–100 W. Of note, the slower kinetics of CO during 50–100 W than 0–50 W (see τ_1_ values in Table 2) may leave the door open to the simultaneous contribution of slower neural and/or mechanical effects to the CO increase.

### Baroreflex sensitivity dynamics

During 0–50 W, MAP decreased abruptly. This was interpreted as being a consequence of the muscular vasodilation (Rådegran and Saltin 1998; Saltin et al. 1998) sustaining the increase in muscle blood flow at exercise onset (Ferretti et al. 1995; DeLorey et al. 2003; Clifford 2007; Chin et al. 2010). In fact, this MAP decrease was coupled with a fast decrease in TPR (Fig. 2f), in line with previous findings (Wieling et al. 1996; Elstad et al. 2002; Lador et al. 2006, 2008). During this MAP decrease, also RRi consensually decreased, as in a baroreflex relationship. This was interpreted as a baroreflex attempt to correct for the MAP fall at exercise onset (Bringard et al. 2017) and, indeed, we identified a long baroreflex sequence upon which it was possible to compute the BRS. As expected, the BRS measured during this initial fall in MAP was lower than that computed during the preceding resting steady state and similar to that measured during the successive 50 W steady state. This is in line with previous observations by Bringard et al. (Bringard et al. 2017), who put forward the hypothesis that the immediate fall of BRS upon exercise start may be due to vagal withdrawal. However, open-loop studies of baroreflexes at exercise steady state showed that the maximal baroreflex gain, measured around the centring point of a baroreflex logistic curve, does not change with exercise (Potts et al. 1993; Norton et al. 1999; Fadel et al. 2001; Ogoh et al. 2003, 2005; Raven et al. 2006). Nevertheless, some authors (Potts et al. 1993; Norton et al. 1999; Fadel et al. 2001; Ogoh et al. 2003, 2005; Raven et al. 2006) reported also a progressive shift of the OP toward the threshold of the open loop baroreflex relationship, which is more marked, the higher the workload. Moreover, Ogoh et al. (Ogoh et al. 2005) found that this OP shift toward the threshold at exercise is present both in control conditions and during sympathetic blockade with metoprolol. This suggests that this OP displacement is sustained by a mechanism acting through modulation of the vagal activity. Since, the BRS corresponds to the baroreflex gain around the OP, an OP shift toward the threshold, i.e., toward a flatter portion of the baroreflex curve, is coherent with the BRS decrease found in this and in a previous study (Bringard et al. 2017). This being so, both the open-loop and the closed-loop findings appear compatible with the hypothesis that vagal withdrawal is the cause of the BRS decrease at exercise. The closed-loop approach made it possible to identify that the BRS decrease upon 0–50 W occurs immediately at exercise start. Thus, we cannot exclude that a central command mechanism may be at the origin of vagal withdrawal. Moreover, since also the initial HR response is extremely rapid, and is likely sustained by vagal withdrawal (Fontolliet et al. 2021), the same mechanism may well cause both the BRS fall and the initial HR increase.

During 50–100 W, which is the most significant and novel aspect of the present study, the abrupt decrease in MAP, which occurred in 0–50 W, was not observed (Fig. 2e). This reflects a slower decrease in TPR (Fig. 2f) and, possibly, a slower regulation of the increase in muscle blood flow upon workload increase in 50–100 W than in 0–50 W. The analysis of the MAP-RRi dynamic relationship revealed a progressive shift toward the successive 100 W steady-state OP (Fig. 4a).

This analysis showed that at the time of the first sequence (6.4 ± 5.5 s after the workload change) the BRS was still similar to that computed during the previous 50 W steady state. Nonetheless, the BRS of the second sequence resulted lower than the BRS at 50 W and similar to that at 100 W steady state. This suggests that the BRS adjustment was slower in this case than during 0–50 W. If the decrease of the BRS around the OP is due to vagal withdrawal, then a further withdrawal of vagal tone may determine (1) the decrease in BRS and (2) the HR phase I response to workload increase also in 50–100 W, yet with slower kinetics.

### Operating point dynamics

In addition to the shift of the OP toward the threshold, as previously discussed, when passing from rest to exercise and with increasing workload, the open-loop analysis also showed that the entire baroreflex relationship moves toward higher values of blood pressure and lower values of RRi (Potts and Raven 1995; Ogoh et al. 2005; Raven et al. 2006). This last baroreflex relationship displacement was defined as baroreflex resetting. Coherently, in the present closed-loop analysis, the OP measured at steady state (Table 1) was characterised by higher MAP values and lower RRi values at 50 W than at rest and at 100 W than at 50 W, indicating progressive displacement of the entire baroreflex relationship downward and leftward. However, at variance with the previously cited studies, we were also able to observe the dynamic displacement of the OP during the two investigated transients, which is precluded with open-loop approach.

During 0–50 W, the OP showed a two-phase movement (Fig. 3a): (1) a decrease in MAP and RRi following a baroreflex relationship until the minimum MAP value was reached, followed by (2) a continuous decrease in RRi coupled with an increase in MAP. The former started at exercise onset and finished at the minimum MAP value, thus lasting 7.9 ± 3.5 s, the latter started at minimum MAP and ended when approaching the 50 W steady state. Our interpretation is that the first phase of the OP displacement may reflect the shift of the OP toward the threshold of the resting baroreflex curve, whereas the second phase may represent the resetting of the entire baroreflex response curve.

During 50–100 W, a baroreflex resetting took place, since both MAP and RRi were, respectively, higher and lower at 100 W than at 50 W steady states (Table 1). However, the OP did not show a two-phase pattern as during its 0–50 W counterpart (Fig. 4a). The baroreflex resetting, in fact, appeared as a progressive displacement of the OP from the 50 W to the 100 W steady state. We propose that this is a consequence of the lack of the sudden large fall in TPR in 50–100 W, and thus of the lack of sudden MAP fall at the beginning of the exercise transient.

### Methodological consideration

In the investigation of the arterial baroreflex, two approaches can be adopted: the open-loop and the closed-loop approaches. In both cases, the HR or blood pressure response (output) to some stimuli capable to activate the arterial baroreflex (input) is recorded and analysed. However, the techniques adopting an open-loop approach assume that any output cannot serve as an input, whereas those adopting a closed-loop approach accept that any output, in terms of HR or blood pressure, is capable to influence the arterial baroreflex and hence to serve as an input.

The currently available open-loop methods, e.g., the neck suction-neck pressure techniques or the use of vasoactive drugs, may provide a more complete description of the baroreflex response both at rest and at exercise. However, their application requires strict steady-state conditions. Therefore, they cannot be applied during the exercise transients. Hence, if we would like to investigate the dynamics of baroreflex readjustments upon exercise start, we are obliged to use a closed-loop approach. Previous literature showed that a modified sequence method can be utilised to this aim (Bringard et al. 2017; Taboni et al. 2021b, a).

## Conclusions

For the first time, the dynamics of the baroreflex adjustments during an exercise transient from light-to-moderate intensity domain have been investigated and compared to a rest-to-light exercise transient. The results showed that during the exercise-to-exercise, with respect to the rest-to-exercise transient, the spontaneous baroreflex sensitivity decreased more slowly and the operating point displacement was not characterised by an initial prompt pressure decrease. Moreover, data suggest that minimal vagal activity is still present in the light intensity domain, and that the withdrawal of the vagal tone could be responsible for the further decrease of the baroreflex sensitivity around the operating point in the transition from light to moderate exercise. The same mechanism may explain also the further rapid increase of heart rate.

Overall, this study represents a step forward in understanding how the arterial baroreflex readjusts to exercise. This understanding appears crucial to explain the exercise intolerance in those conditions characterised by arterial baroreflex impairment, as in spinal cord injuries, Parkinson’s disease, or hypertension. In this context, this study set the basis for further investigations aimed at developing countermeasures to exercise and orthostatic intolerance in those patients coping with blood pressure dysregulation.

## Supplementary Information

Below is the link to the electronic supplementary material.

Supplementary file1 (XLSX 12 KB)

Supplementary file2 (XLSX 12 KB)

## Data Availability

The datasets generated and analysed during the current study are available from the corresponding author on reasonable request.

## List of symbols

0–50 W: Rest-to-50 W exercise transients
50–100 W: 50-to-100 W exercise transients
*A*_1_: Amplitude of the initial phase of the cardiovascular responses
*A*_2_: Amplitude of the primary phase of the cardiovascular responses
*b*: Baseline
BRS: Spontaneous baroreflex sensitivity
CO: Cardiac output
CO_I_: Cardiac output asymptote of the initial phase
*d*: Time delay between the initial and the primary phases of the cardiovascular responses
DAP: Diastolic arterial pressure
HR: Heart rate
MAP: Mean arterial pressure
OP: Operating point
RRi: R-to-R interval
SAP: Systolic arterial pressure
SV: Stroke volume
TPR: Total peripheral resistances
τ_1_: Time constant of the initial phase of the cardiovascular responses
τ_2_: Time constant of the primary phase of the cardiovascular responses
[La]: Blood lactate concentration
